# Efficacy of radical doses of pelvic radiotherapy for primary tumor treatment in patients with newly diagnosed organ metastatic cervical cancer

**DOI:** 10.1186/s13014-019-1297-x

**Published:** 2019-05-20

**Authors:** Zhuomin Yin, Hanmei Lou, Huarong Tang, Juan Ni, Qiong Zhou, Ming Chen

**Affiliations:** 1 Department of Radiation Oncology, The Second Affiliated Hospital of Soochow University, Suzhou, China; 2 Department of Gynecologic Radiation Oncology, Zhejiang Cancer Hospital, Hangzhou, China; 3 Department of Radiation Oncology (Zhejiang Key Laboratory of Radiation Oncology), Zhejiang Cancer Hospital, No. 1 Banshan East Road, Hangzhou, 310022 China

**Keywords:** Uterine cervical neoplasms, Organ metastasis, Primary tumor treatment, Definitive, Radiotherapy

## Abstract

**Background:**

The clinical efficacy of definitive pelvic radiotherapy for primary tumors in patients with newly diagnosed organ metastatic cervical cancer is unclear. Therefore, we conducted a retrospective study to evaluate the efficacy of definitive pelvic radiotherapy combined with systemic chemotherapy in patients with organ metastatic cervical cancer.

**Methods:**

We retrospectively analysed medical records from patients with newly diagnosed organ metastatic cervical cancer, all treated with chemotherapy at the Zhejiang Cancer Hospital between October 2006 and December 2016. Survival times were compared using the Kaplan-Meier method. The univariate log-rank method and multivariate Cox proportional hazard models were used to identify associated variables with survival.

**Results:**

A total of 48 patients were identified from 11,982 primary cervical cancer patients and divided into two groups according to treatment mode: 36 patients received chemotherapy combined with definitive pelvic radiotherapy (group A), 12 patients underwent chemotherapy with/without palliative pelvic radiotherapy (group B). Median follow-up was 14.4 months (range, 4.6–114.7 months). Median overall survival (OS) for group A and group B was 17.3 and 10 months, respectively. Using the univariate analysis, group A was found to have a better OS than group B (*p* = 0.002). In multivariate analysis, group A (hazard ratio [HR], 0.32; 95% confidence interval [CI], 0.15–0.67, *p* = 0.003) was associated with lower risk of death compared with group B. The main reason for treatment failure was found to be due to the progression of distant metastatic lesions in 36 patients (75%) from the whole cohort.

**Conclusion:**

In this cohort of organ metastatic cervical cancer patients in good performance status, chemotherapy combined with definitive pelvic radiotherapy was associated with improved survival outcomes when compared with chemotherapy with/without palliative pelvic radiotherapy. Prospective trials evaluating definitive pelvic radiotherapy for newly diagnosed organ metastatic cervical cancer, therefore, are warranted.

## Background

Newly diagnosed organ metastatic cervical cancer is defined as a cervical carcinoma that has extended to distant organs such as the lung, bone, liver or brain. Approximately 3% of patients have the International Federation of Gynecology and Obstetrics (FIGO) stage IVB cervical cancer, and patients with organ metastasis account for only a small proportion of these [1].

For locally advanced cervical cancer, definitive pelvic radiotherapy combined with concurrent chemotherapy has been employed as a standard treatment modality [2]. However, the role of definitive pelvic radiotherapy for primary tumor treatment in patients with organ metastatic cervical cancer at presentation is unclear. Systemic treatment based on combination chemotherapy is recommended by the guidelines of the National Comprehensive Cancer Network (NCCN) and the European Society of Gynecological Oncology (ESGO) [3, 4]. Currently, combination chemotherapy alone is widely used as a treatment for cervical cancer patients with organ metastasis, but with limited benefit and poor response outcomes [5–7].

Increasing evidence suggests that local radiotherapy may be related to improved survival in patients with some specific types of metastatic cancers [8–10]. Therefore, we performed a retrospective study to evaluate the efficacy of definitive pelvic radiotherapy based on chemotherapy in patients with newly diagnosed organ metastatic cervical cancer.

## Methods

### Patients

The present study was a single-institutional retrospective analysis of 48 patients with organ metastasis who were identified from 11,982 newly diagnosed primary cervical cancer patients between October 2006 and December 2016 at Zhejiang Cancer Hospital, in Hangzhou, China. Approval for this study was obtained from the Institutional Review Board and Ethics Committee of the Zhejiang Cancer Hospital (No. IRB-2017-94).

Medical records of patients initially diagnosed with organ metastatic cervical cancer were reviewed. The inclusion criteria were as follows: biopsy-confirmed cervical cancer, International Federation of Gynecology and Obstetrics (FIGO) stage IVB with organ metastasis, received at least 2 cycles of chemotherapy plus or minus radiotherapy, with an Eastern Cooperative Oncology Group (ECOG) performance status score of 2 or less, and life expectancy > 6 months. Patients for whom cervical cancer was not the first malignancy or with incomplete clinical data were excluded.

Organ metastatic cervical cancer was defined as cervical cancer hematogenous metastases to bone or visceral organs (lung, liver, brain). Metastatic lesions were diagnosed mainly on the basis of the following examinations: firstly conventional imaging procedures, such as magnetic resonance imaging (MRI), computed tomography (CT) and emission computed tomography (ECT), secondly positron emission tomography-computed tomography (PET-CT), and finally histopathological or cytopathological biopsy, with the proportion being 47.9, 39.6, and 12.5%, respectively. In principle, pathological confirmation is necessary for metastatic lesions, but the biopsy is invasive and risky. Therefore the lesions of organ metastasis were identified by biopsy only when they could not be identified by imaging. Lymph node > 10 mm enlargement in the short axis based on CT represented defined lymph node metastasis. All examinations were carried out at the discretion of the treating physician.

### Treatment

Depending on its role, radiotherapy can be divided into local definitive radiotherapy, or palliative radiotherapy. It can also be classified into the primary tumor or metastatic lesion radiotherapy according to different radiation sites. In this study, definitive pelvic radiotherapy for primary tumors was used including external beam radiation therapy (EBRT) and high dose-rate intracavitary brachytherapy (BT). EBRT for primary pelvic tumors was delivered using 4-field box, 3D-conformal radiation therapy (3D-CRT) or intensity modulated radiation therapy (IMRT) to a median dose of 45.9 Gy (range 45–50.4 Gy) in 1.7–1.8 Gy per fraction. BT using an iridium-192 source was carried out after completion of the EBRT to a median dose of 28 Gy (range 23–34.5 Gy) in 4–6 Gy per fraction. Four patients received pelvic EBRT with central shielding, while most patients received EBRT with no central shielding. Metastatic lymph nodes in the pelvic or para-aortic region were simultaneously or sequentially boosted to a total dose of 54–60 Gy. Besides, palliative EBRT for the primary pelvic disease was performed in five patients to a median dose of 37.8 Gy (range 10.8–46.8 Gy) in 1.7–1.8 Gy per fraction.

All patients received at least two cycles of chemotherapy, in the form of a platinum-containing doublet or single-agent cisplatin regimens. Platinum-containing doublet regimens mainly included cisplatin/paclitaxel, carboplatin/paclitaxel and cisplatin/etoposide every 21 or 28 days. The doublet chemotherapy was performed before radiotherapy, concurrently with radiotherapy, or after radiotherapy. Single-agent cisplatin regimen chemotherapy was carried out concurrently with radiotherapy weekly.

### Patient follow-up

After treatment, patients were generally followed in the clinic or by telephone every 3 months for the first 2 years, then every 6 months for the next 3 years, and annually after that. Disease progression and death were the primary endpoints, and both computed from the date of diagnosis confirmation. Follow-up imaging (CT, MRI, or PET-CT) and pathological examinations were used to evaluate disease progression. Progression had to be confirmed by at least two experts, including radio-oncologists, imaging specialists, and pathologists. The date of imaging or pathology for confirmation of the recurrence was defined as the date of progression. Overall response evaluation was performed by the Response Evaluation Criteria in Solid Tumors (RECIST) version 1.1, measured by CT, MRI or PET-CT.

### Statistics

The endpoints included overall survival (OS) and progress-free survival (PFS). The potential associations between various factors (age, histological subtype, size of the cervical tumor, ECOG score, pre-treatment haemoglobin (Hb) level, pre-treatment white blood cell count (WBC) level, number and site of organ metastasis, distant lymph node metastasis and treatment mode) and survival outcomes were assessed. The survival curves were compared, and univariate analyses were performed to identify the prognostic factors with the log-rank test of Kaplan-Meier. Cox regression analyses were performed to identify the independent prognostic factors and establish the hazard ratio (HR) for PFS and OS by multivariate analyses. Values of *p* < 0.05 were considered statistically significant. All statistical analyses were conducted with SPSS statistical software (version 22.0; IBM Corp., Armonk, NY, USA).

## Results

### Patient characteristics

A total of 48 patients were enrolled, and their characteristics before treatment are presented in Table 1. The median age was 53 years (range 36–77 years), and the most common histological subtype was squamous cell carcinoma in 39 patients (81.3%). An ECOG score for all patients was in the range of 0 to 2. All patients received a total of 213 cycles of chemotherapy, and the median 4 cycles ranged from 2 to 8 for each patient. The most frequent chemotherapy regimen was cisplatin/paclitaxel (156, 73.2%), cisplatin/etoposide (20, 9.4%), single-agent cisplatin (18, 8.5%), and carboplatin/paclitaxel (10, 4.7%).

**Table 1:** Patient baseline characteristics at the time of initial diagnosis and treatment types (*n* = 48)

Characteristics	Median (range)/no. of patients (%)		
	Total	Group A	Group B
No. of patients	48	36	12
Age (yr), median (range)	53 (36–77)	54 (37–68)	47 (36–77)
ECOG performance status score			
0	33 (68.8)	26 (72.2)	7 (58.3)
1	11 (22.9)	7 (19.4)	4 (33.3)
2	4 (8.3)	3 (8.3)	1 (8.3)
Histological subtype			
Squamous cell carcinoma	39 (81.3)	32 (88.9)	7 (58.3)
Small-cell neuroendocrine carcinoma	4 (8.3)	1 (2.8)	3 (25.0)
Adenocarcinoma	3 (6.3)	2 (5.6)	1 (8.3)
Adeno-squamous cell carcinoma	1 (2.1)		1 (8.3)
Sarcoma	1 (2.1)	1 (2.8)	
Primary tumor size (cm)			
≥ 4-cm	39 (81.3)	30 (83.3)	9 (75.0)
< 4-cm	9 (18.8)	6 (16.7)	3 (25.0)
Pre-treatment Hb (g/dL)			
> 10	37 (77.1)	29 (80.6)	8 (66.7)
≤ 10	11 (22.9)	7 (19.4)	4 (33.3)
Pre-treatment WBC(10^9 /L)			
> 10	8 (16.7)	6 (16.7)	2 (16.7)
≤ 10	40 (83.3)	30 (83.3)	10 (83.3)
Diagnostic methods of metastatic lesions			
CT/MRI/ECT	23 (47.9)	18 (50.0)	5 (41.7)
PET-CT	19 (39.6)	14 (38.9)	5 (41.7)
Biopsy	6 (12.5)	4 (11.1)	2 (16.7)
Initial treatment			
Chemotherapy cycle	4 (2–8)	4 (2–7)	4 (3–8)
Pelvic primary tumor radiotherapy	42 (87.5%)	36 (100%)	5 (41.7%)
Sites of organ metastasis radiotherapy	19 (39.6%)	17 (47.2%)	2 (16.7%)
Primary tumor radiotherapy dose (Gy)			
External dose		45.9 (45–50.4)	37.8 (10.8–46.8)
ICBT dose		28 (23–34.5)	
Point A dose (EQD2)		80.4 (72.3–87)	

*ECOG* Eastern Cooperative Oncology Group, *Hb* hemoglobin, *WBC* white blood cell count, *MRI* magnetic resonance imaging, *CT* computed tomography, *ECT* emission computed tomography, *PET-CT* positron emission tomography-computer tomography, *ICBT* intracavitary brachytherapy, *EQD2* equivalent dose in 2 Gy fractions

Patients were divided into two groups depending on whether they had been given definitive pelvic radiotherapy. Patients in group A received chemotherapy combined with definitive pelvic radiation therapy (*n* = 36), and patients in group B received chemotherapy only, or chemotherapy combined with palliative pelvic radiotherapy (*n* = 12). The median equivalent dose in 2 Gy fractions (EQD2) of 80.4Gy (range 72.3-87Gy) was delivered to the patients in group A. Palliative radiotherapy at sites of organ metastasis was performed in 17 patients (47.2%) in group A. Of the 36 patients in group A, 20 (55.6%) received neoadjuvant chemotherapy, 18 patients (50%) received concurrent chemotherapy and 17 patients (47.2%) received adjuvant chemotherapy. For group B, five patients (41.7%) received palliative pelvic radiotherapy, and two patients (16.7%) received palliative radiotherapy at sites of organ metastasis (Table 1).

The distribution of the distant metastases is shown in Table 2. Among a total of 59 organ metastatic sites identified, the most common sites were bone (28, 47.5%), lung (14, 23.7%) and liver (10, 16.9%). Among all patients, 21 (43.8%) displayed oligometastases (limited to a single organ less than or equal to three metastases), 37 patients (77.1%) had single organ metastasis, and 20 patients (41.7%) were combined with distant (extra-pelvic) lymph node metastasis.

**Table 2:** Distribution of distant metastasis for 48 patients with cervical cancer

Sites of distant metastasis	No. of patients (%)
Sing organ metastasis	
Bone	17 (35.4)
Lung	5 (10.4)
Liver	1 (2.1)
Brain	1 (2.1)
Spleen	1 (2.1)
Sing organ +distant lymph node metastasis	
Bone+distant lymph node	5 (10.4)
Lung+distant lymph node	4 (8.3)
Liver+distant lymph node	3 (6.3)
Two organ metastasis	
Lung+spleen	2 (4.2)
Bone+liver	1 (2.1)
Two organ metastasis+distant lymph node metastasis	
Liver+lung+distant lymph node	2 (4.2)
Bone+lung+distant lymph node	3 (6.3)
Liver+bone+distant lymph node	2 (4.2)
Liver+adrenal gland+distant lymph node	1 (2.1)

### Survival outcome

The median follow-up period for the whole cohort was 14.4 months (range 4.6–114.7 months). We found that the median OS time was 14.8 months, with 1, 2, and 5-year OS rates of 60.4, 32.5, and 20.2%, respectively. Median PFS time was 8.3 months, with1, 2, and 5-year PFS rates of 34.1, 24.2, and 19.4%, respectively. Median OS time for group A and group B was 17.3 and 10 months, median PFS time for group A and group B was 9.4 and 4.1 months respectively.

**Fig. 1:**
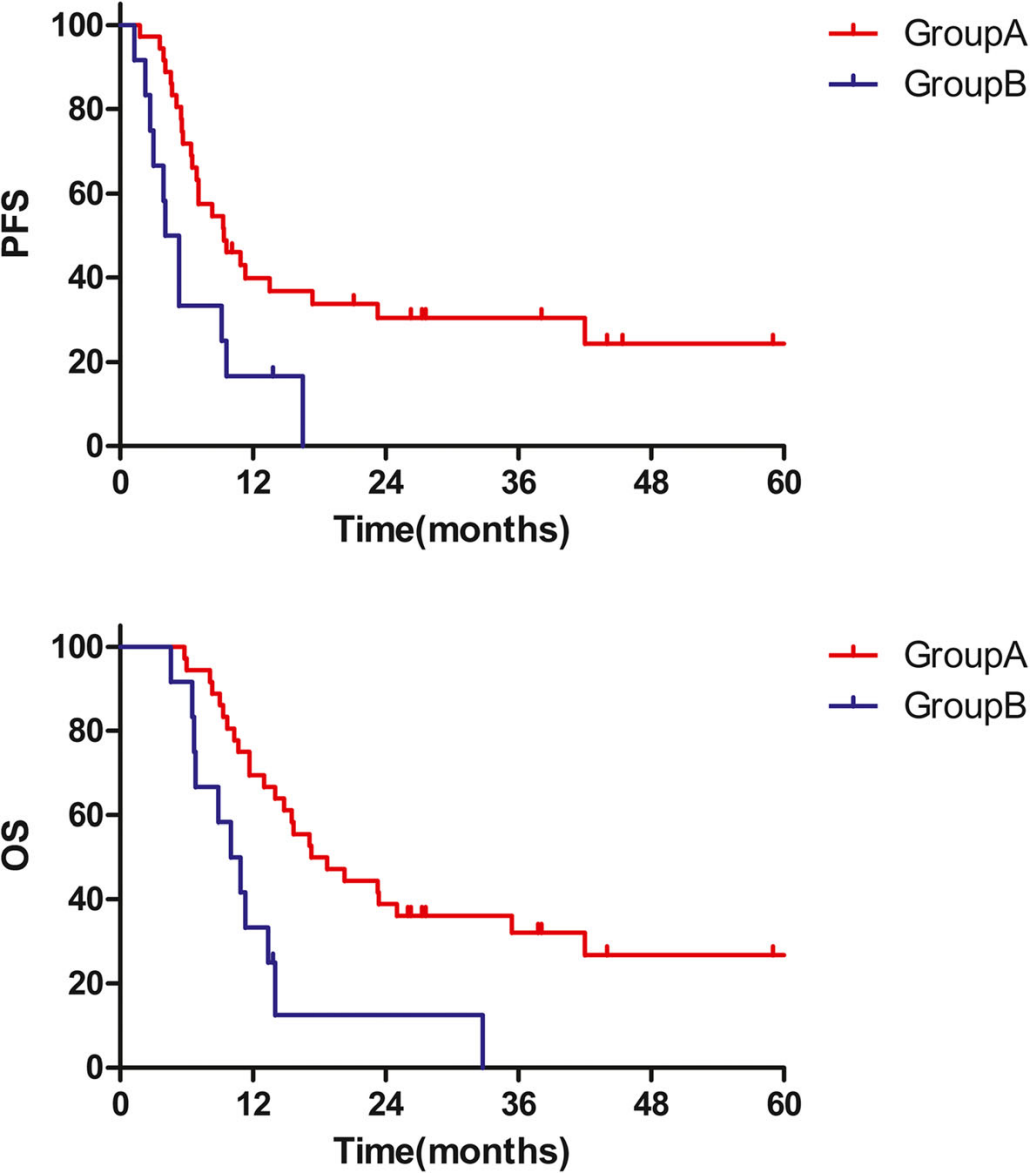
Kaplan–Meier survival curves for patients in the group A and group B. (group A, chemotherapy combined with definitive pelvic radiotherapy; group B, chemotherapy with/without palliative pelvic radiotherapy; PFS, progression-free survival; OS, overall survival)

By using univariate analysis, our results showed in Table 3 that squamous cell carcinoma, oligometastasis and treatment mode of group A were associated with improved OS. Improved PFS was also observed for these patients at age ≥ 50 years, for oligometastasis and treatment mode of group A. The two-year OS rates for group A were better than those for group B (38.9% vs. 12.5%, *p* = 0.002) and the two-year PFS rates for group A were also better than those for group B (30.4% vs. 0%, *p* = 0.008) (Fig. 1).

**Table 3:** Univariate and multivariate analysis of prognostic factors for progression-free survival and overall survival (*n* = 48)

Variable	No. of patients	Univariate analysis				Multivariate analysis			
		2-y Survival rate (%)				PFS		OS	
		PFS	*p*-value	OS	*p*-value	HR (95% CI)	*p*-value	HR (95% CI)	*p*-value
Age									
≥ 50 years	29	35.6		36.8		0.52 (0.27–1.01)	0.053		
< 50 years	19	10.5	0.034	26.3	0.096	Reference			
Size of the cervical tumor									
≥ 4-cm	39	21.7		28.2					
< 4-cm	9	38.1	0.338	51.9	0.207				
Histological subtype									
Squamous cell carcinoma	39	28.0		38.5					
Non-squamous cell carcinoma	9	11.1	0.064	0	0.002				
ECOG performance status score									
0	33	27.6		35.2					
1–2	15	16.7	0.466	26.7	0.654				
Pre-treatment Hb (g/dL)									
> 10	37	23.1		29.7					
≤ 10	11	27.7	0.622	41.6	0.385				
Pre-treatment WBC(10^9 /L)									
> 10	8	25.0		25.0					
≤ 10	40	23.8	0.415	34.0	0.330				
Oligometastasis									
Yes	21	37.5		52.4					
No	27	12.2	0.037	16.3	0.042				
Bone metastasis									
Yes	28	23.1		33.9					
No	20	25.5	0.716	30	0.662				
Number of organ metastasis									
Single	37	26.7		37.8					
Multiple	11	18.2	0.371	12.1	0.160				
Lung metastasis									
Yes	14	8.2		14.3					
No	34	30.3	0.116	40	0.115				
Liver metastasis									
Yes	10	24.3		25					
No	38	26.7	0.805	34.2	0.656				
Distant lymph node metastasis									
Yes	20	16		16.9					
No	28	29.5	0.203	42.9	0.063				
Treatment mode									
Group A	36	30.4		38.9		0.40 (0.19–0.84)	0.015	0.32 (0.15–0.67)	0.003
Group B	12	0	0.008	12.5	0.002	Reference		Reference	

*ECOG* Eastern Cooperative Oncology Group, *CI* confidence interval, *HR* hazard ratio, *OS* overall survival, *PFS* progression-free survival, *Hb* hemoglobin, *WBC* white blood cell count

The results of the multivariate analyses revealed that only those patients in group A receiving definitive pelvic radiotherapy combined with chemotherapy (hazard ratio [HR], 0.32; 95% confidence interval [CI], 0.15–0.67, *p* = 0.003) as an independent prognostic factor was related to a reduction in death (Table 3). Of note, based on the multivariate analysis results, we also established that only patients in group A who underwent definitive pelvic radiotherapy combined with chemotherapy (HR, 0.40; 95% CI, 0.19–0.84, *p* = 0.015) as an independent prognostic factor were associated with a 60% lower risk of being progression (Table 3).

### Patterns of progression

At the time of analysis, a total of 38 patients (79.2%) experienced disease progression, as determined by distant metastatic lesion progression (36, 75%), followed by regional pelvic progression (4, 8.3%), with two patients experiencing both. The median time of progression was 7.1 months. At the end of the follow-up, 12 patients (25%) were still alive, and 36 patients (75%) were deceased due to disease progression (Table 4).

**Table 4:** Outcomes and patterns of progression in the different group

Group	CR (%)		PR (%)	SD (%)	PD (%)		D(%)
	Locoregional	Distant			Locoregional	Distant	
Group A (*n* = 36)	27 (75)	1 (2.8)	7 (19.4)	1 (2.8)	2 (5.6)	27 (75)	25 (69.4)
Group B (*n* = 12)	0 (0)	0 (0)	1 (8)	0 (0)	2 (17)	9 (75)	11 (91.7)
Overall (*n* = 48)	27 (56.3)	1 (2.1)	6 (12.5)	0 (0)	4 (8.3)	36 (75)	36 (75)

*CR* Complete remission, *PR* Partial remission, *SD* Stable disease, *PD* Progressive disease, *D* death

In group A, 27 patients (75%) achieved pelvic locoregional complete remission through definitive pelvic radiotherapy combined with chemotherapy. The leading cause of failure was distant metastatic lesion progression in 27 patients (75%); among these, two patients simultaneously developed regional pelvic failure. Of the 12 patients in group B, one patient (8.3%) survived with partial remission, the remaining 11 patients (91.7%) underwent disease progression, among these, nine patients (75%) with distant metastatic lesions progression and two patients (16.7%) with regional pelvic progression (Table 4).

## Discussion

In this study, we have attempted to assess the efficacy of definitive pelvic radiotherapy combined with chemotherapy in patients with organ metastatic cervical cancer. Our results demonstrated that chemotherapy combined with definitive pelvic radiotherapy improved survival outcome compare with chemotherapy with/without palliative pelvic radiotherapy. The pelvic local control rate was high for patients receiving the definitive pelvic radiotherapy combined with chemotherapy. However, 75% of patients still experienced failure with distant metastatic lesion progression.

Patients with newly diagnosed organ metastatic cervical cancer experienced a poor prognosis [11–13]. At present, the generally accepted treatment is combinational chemotherapy-based systemic therapy, whereas the role of definitive pelvic radiotherapy for primary tumor as a local treatment is unclear. In the ESGO guideline, combination chemotherapy (cisplatin/paclitaxel and carboplatin/paclitaxel) is recommended while the central role of radiotherapy is palliative, to control pain and bleeding in patients with organ metastasis at diagnosis [4]. In the NCCN guidelines, depending on whether the disease is amenable to local treatment, two treatment modalities have been recommended. However, the criteria for local treatment adaptation have not yet been clearly defined [3]. For patients with organ metastatic cervical cancer, it is still unclear as to the benefits of active local treatment combined with chemotherapy.

As methods for local treatment, surgery and radiotherapy are all recommended in the 2019 NCCN guideline for distant metastatic cervical cancer [3]. Surgical resection treatment can be a useful treatment for lesions of distant metastases [14]. However, as far as primary uterine cervical tumors are concerned, radiotherapy is more suitable than surgical treatment, as most patients with organ metastatic cervical cancer have locally advanced disease.

In our study, most of the treatment failures due to distant progress; people wondered whether definitive pelvic radiotherapy as a local treatment method is a reasonable choice for women with organ metastatic cervical cancer. At present, there is a growing body of evidence supporting a beneficial role for definitive radiotherapy in the sites of primary or metastatic tumors. A large-sample (3169 patients) retrospective study on newly diagnosed metastatic cervical cancer showed that patients who received chemotherapy alone, EBRT alone plus chemotherapy, or EBRT/BT plus chemotherapy had a median survival time 10.1, 12.9, and 27.5 months, respectively; of note, chemoradiotherapy was associated with improved survival than chemotherapy alone for patients with organ metastasis by subgroup analyses (*p* < 0.05) [15]. Another significant (6382 cases) retrospective study of newly diagnosed metastatic prostate cancer found that patients undergoing prostate radiotherapy and androgen deprivation survived substantially longer than patients receiving androgen deprivation alone; meanwhile, improved overall survival was observed for higher-dose radiotherapy (≥ 65Gy) when compared with lower-dose radiation therapy (< 65 Gy) by subgroup analyses [9]. In addition, some studies showed chemotherapy combined with definitive radiotherapy to sites of oligometastatic diseases achieved favorable survival rates, and the toxicity of radiotherapy-related was minimal and well tolerated [8, 10]. A similar trend was identified in our study, patients with organ metastatic cervical cancer receiving systemic chemotherapy combined with definitive pelvic radiotherapy had increased survival rates when compared with patients receiving systemic chemotherapy with/without palliative pelvic radiotherapy. All these evidence support the use of systemic chemotherapy combined with definitive pelvic radiotherapy for certain women with organ metastatic cervical cancer in good performance status.

In addition to survival benefits, there have been some reports of chemoradiotherapy for cervical cancer with organ metastases giving potential insights into the patient selection. A retrospective study of 24 patients with stage IVB cervical cancer (87.5% with organ metastases) receiving radiotherapy and multiagent chemotherapy found that improved 5-year OS rates (22%) were observed among patients with good performance status [16]. An additional retrospective study looking at prognosis factors from 111 patients with stage IVB cervical cancer has indicated that performance status is one of the independent prognostic factors [13]. In our study, all patients with a good ECOG performance status score had a good tolerance potential for radiotherapy and chemotherapy and showed a 5-year OS rate of 20.2%.

Although high pelvic local control rates were achieved for patients receiving definitive pelvic radiotherapy, distant metastatic lesion progression was the main reason for treatment failure. Our study showed that in group A, 27 patients, representing 75% achieved local complete remission from pelvic primary tumors utilizing definitive pelvic radiotherapy combined with chemotherapy, yet 27 patients (75%) developed treatment failure for distant lesion progression. This result was supported by findings of an earlier study retrospectively analysing 50 patients diagnosed with disseminated cervical cancer with distant lymph nodal or visceral organ metastasis and found that the primary reason for treatment failure was the systemic progression in 32 patients (64%) [17]. Therefore, systemic therapy for organ metastatic cervical cancer is still the most important treatment.

A phase III Gynecologic Oncology Group (GOG) study found that chemotherapy combined with bevacizumab treatment compare with chemotherapy alone significant improved OS (16.8 vs 13.3 months) for patients with recurrent/persistent/metastatic cervical cancer [18]. Therefore, standard chemotherapy (cisplatin/paclitaxel and carboplatin/paclitaxel) combined with bevacizumab is recommended for patients with a good performance status [4]. Recently, an immune checkpoint inhibitor, programmed cell death protein-1 (PD-1), and programmed cell death ligand-1 (PD-L1), showed potential for the treatment of advanced cervical cancer [19]. These new drugs also bring new hope and new research directions for the systemic treatment of organ metastatic cervical cancer.

For patients with organ metastatic cervical cancer in good performance status, systemic treatment (chemotherapy, molecular targeting therapy, and immunotherapy) should be administered first. When systemic treatment is effective, radiotherapy should be considered for local treatment. For some patients with good prognostic factors, such as a limited number of metastases, a definitive radiation dose (EBRT plus BT) for the primary cervical tumor should be considered. Although a few patients with favorable prognostic factors might be completely cured by chemoradiotherapy, some patients may achieve long-term survival with neoplasm. For patients with unfavorable prognostic factors, the aim of definitive pelvic radiotherapy may be to gain good local control, improve quality of life and further delay the progression.

The major limitations of this study are the following: firstly, the inherent bias in a single-institution retrospective study, the small sample size, and covering a long period. Secondly, heterogeneity within patient groups and treatments might represent confounding factors that may influence the outcome. Thirdly, the decision of which treatment regimen should be adopted for a patient may be influenced by a variety of factors, including the opinions from doctor’s team and the decision from patient’s own; furthermore, doctor’s judgment may be affected by patient’s tolerability and efficacy of previous treatment. Fourthly, although survival was the only endpoint assessed here, information on other factors such as quality of life, and symptom control are also important, but these are not found in the database. To eliminate these potential biases, we should conduct prospective multicenter clinical studies in the future.

## Conclusion

Despite the inherent drawbacks of retrospective analyses, our data indicate that patients with newly diagnosed organ metastatic cervical cancer, but in good performance status may benefit from definitive pelvic radiotherapy combined with chemotherapy to improve overall survival, especially for some patients with favorable prognostic factors. Prospective multicenter clinical trials to further validate the findings of this study are warranted.

## Abbreviations

3D-CRT: 3D-conformal radiation therapy
CI: Confidence interval
CR: Complete remission
CT: Computed tomography
D: Death
EBRT: External beam radiotherapy
ECOG: Eastern Cooperative Oncology Group
ECT: Emission computed tomography
EQD2: Equivalent dose in 2 Gy fractions
ESGO: European Society of Gynecological Oncology
FIGO: International Federation of Gynecology and Obstetrics
GOG: Gynecologic Oncology Group
Hb: Haemoglobin
HR: Hazard ratio
ICBT: Intracavitary brachytherapy
IMRT: Intensity modulated radiation therapy
MRI: Magnetic resonance imaging
NCCN: National Comprehensive Cancer Network
OS: Overall survival
PD: Progressive disease
PD-1: Programmed cell death protein-1
PD-L1: Programmed cell death ligand-1
PET-CT: Positron emission tomography-computed tomography
PFS: Progress-free survival
PR: Partial remission
RECIST: Response Evaluation Criteria in Solid Tumors
SD: Stable disease
WBC: White blood cell count
